# Differential impact of diabetes mellitus on antiplatelet effects of prasugrel and clopidogrel

**DOI:** 10.1186/s12959-017-0159-8

**Published:** 2018-03-15

**Authors:** Satoshi Niijima, Tsukasa Ohmori, Kazuomi Kario

**Affiliations:** 1 Division of Cardiovascular Medicine, Department of Medicine, Jichi Medical University School of Medicine, 3311-1 Yakushiji, Shimotsuke, Tochigi 329-0498 Japan; 2 Department of Biochemistry, Jichi Medical University School of Medicine, 3311-1 Yakushiji, Shimotsuke, Tochigi 329-0498 Japan

**Keywords:** Thienopyridines, P2Y_12_, Vasodilator-stimulated phosphoprotein phosphorylation

## Abstract

**Background:**

Although prasugrel exerts stronger antiplatelet effects compared with clopidogrel, the factors affecting platelet reactivity under prasugrel have not been fully determined. This study aimed to find the novel mechanistic differences between two thienopyridines and identify the factor that influence platelet reactivity to each drug.

**Methods:**

Forty patients with stable angina who underwent elective percutaneous coronary intervention were randomly assigned to receive either prasugrel (20 mg) or clopidogrel (300 mg) as a loading dose. Platelet function (light transmission, laser light scattering, and vasodilator-stimulated phosphoprotein phosphorylation) and plasma active metabolite levels were measured after the loading dose.

**Results:**

Prasugrel consistently inhibited adenosine diphosphate receptor P2Y_12_ signalling to abolish amplification of platelet aggregation. Prasugrel abolished even small platelet aggregates composed of less than 100 platelets. On the other hand, clopidogrel inhibited large aggregates but increased small and medium platelet aggregates. Diabetes was the only independent variable for determining antiplatelet effects and active metabolite concentration of prasugrel, but not clopidogrel. Sleep-disordered breathing was significantly correlated with platelet reactivity in patients who had clopidogrel.

**Conclusions:**

Prasugrel efficiently abolishes residual P2Y_12_ signalling that causes small platelet aggregates, but these small aggregates are not inhibited by clopidogrel. Considering the differential effect of diabetes on antiplatelet effects between these two drugs, the pharmacokinetics of prasugrel, other than cytochrome P450 metabolism, might be affected by diabetes.

**Trial registration:**

UMIN-CTR UMIN000017624, retrospectively registered 21 May 2015.

## Background

Dual antiplatelet therapy with aspirin and thienopyridine is a common therapy in patients who are undergoing percutaneous coronary intervention (PCI) to protect stent thrombosis [1, 2]. Clopidogrel is broadly used to inhibit the adenosine diphosphate (ADP) receptor P2Y_12_, but it shows a delayed onset of action and a great inter-individual variability of drug efficacy [3, 4]. Absorbed clopidogrel is converted to an active metabolite through two oxidation steps by hepatic cytochrome P450 (CYP) [5]. High on-treatment platelet reactivity under clopidogrel therapy because of genetic polymorphisms in *CYP2C19* and drug-interactions increases the risk of cardiovascular events after PCI [3, 4]. These limitations of clopidogrel have led to the development of alternative P2Y_12_ inhibitors with a rapid onset of action and consistent antiplatelet activity.

Prasugrel is a new-generation thienopyridine that shows more potent inhibition of P2Y_12_ with rapid onset of action, and significantly reduces ischaemic events after PCI compared with clopidogrel [6, 7]. Prasugrel is effectively converted to an active metabolite by esterases followed by a single oxidation step [8]. The peak plasma concentration of prasugrel is reached at 30 min after dosing in stable PCI patients, and is not affected by *CYP2C19* polymorphisms, body mass index, diabetes, smoking, and renal impairment [9]. Prasugrel treatment consistently results in fewer poor responders compared with clopidogrel, while inadequate platelet inhibition has also been reported. Platelet reactivity under prasugrel treatment is an independent risk factor for adverse events or bleeding after PCI [10, 11]. However, the factors that affect platelet reactivity under prasugrel treatment have not been fully determined. In this study, we prospectively compared the pharmacodynamics and platelet reactivity of clopidogrel and prasugrel in a randomized trial.

## Methods

### Patient population and study design

This study was a prospective, randomized, open-label, parallel-group, single-centre study that was conducted in Japan between November 2014 and April 2016 (UMIN000017624). The protocol was approved by the Institutional Review Board of Jichi Medical University on 14 October 2014. From the day on, we started to recruit patients, and total 10 patients were included before completion of clinical trial registration. Patients who were scheduled to undergo PCI with suspected angina pectoris were enrolled. Eligible subjects for the study met all of the following inclusion criteria: (1) typical ischaemic symptoms with coronary risk factors or a positive study for coronary computed tomography (CT), myocardial perfusion scintigraphy, or a treadmill exercise test; (2) on treatment with low-dose aspirin (100 mg/day) for at least 7 days; and (3) older than 20 years of age. Exclusion criteria were any of the following: (1) use of any antiplatelet agents other than aspirin, or oral anticoagulant agents within 7 days before admission; (2) body weight of 50 kg or less; (3) a history of active bleeding; (4) any active malignancy or collagen disease; (4) a platelet count of <100,000/mm^3^ or >400,000/mm^3^; (5) participation in other clinical trials; (6) creatinine levels >2.5 mg/dl; and (7) liver disease (bilirubin levels >2 mg/dl). Hypertension was defined as a systemic blood pressure of 140 mmHg or higher or taking any antihypertensive drug. Dyslipidaemia was defined as serum low-density lipoprotein cholesterol levels of 140 mg/dl or higher or taking statins. Diabetes was diagnosed by taking oral hypoglycaemic agents or insulin, or glycosylated haemoglobin (HbA1c) was 6.5% or greater.

**Fig. 1:**
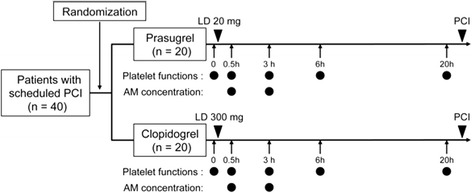
Study design at loading dose. Forty patients who were scheduled for percutaneous coronary intervention (PCI) under treatment with aspirin were randomized at admission to treatment of either a 20-mg loading dose (LD) of prasugrel or a 300-mg LD of clopidogrel. Platelet function analyses were performed at five time points (baseline, and 0.5, 3, 6, and 20 h) after the LD. Active metabolite concentrations were measured at 0.5 and 3 h after the LD

Patients who met all of the criteria listed above were randomized at admission to treatment of either an oral loading dose (LD) of prasugrel 20 mg followed by a 3.75-mg/day maintenance dose or an oral LD of clopidogrel 300 mg followed by a 75-mg/day maintenance dose for 14 days (Fig. 1). The randomization ratio was 1:1 between two treatment arms and assignment was determined with a computer-based table of randomization system by a secretary who was unaware of the study protocol. The same dose of aspirin was continued during the study. Primary outcomes of the study were to compare responses of platelets to the treatment with prasugrel and clopidogrel. Further, this study aimed to identify the factor that influence platelet reactivity to each drug. Here, we analyzed on the responses to LD. Platelet function analyses were performed at five time points (baseline, and 0.5, 3, 6, and 20 h) after the LD (Fig. 1). The concentrations of active metabolites were measured at 0.5 and 3 h after the LD (Fig. 1). Blood was collected before meal mainly. Blood at 3 h after dosing was only collected after meal.

### Platelet function assays and measurement of active metabolites

Blood for platelet function analysis was collected from the antecubital vein using a 21-guage needle into a syringe containing 1/10 sodium citrate at pre-defined time points. Platelet functions were assessed by light transmission and light scattering intensity using a PA-200C platelet aggregation analyser (Kowa Co., Ltd., Tokyo, Japan) [12, 13]. This equipment is particularly sensitive for detecting the size of small platelet aggregates and can classify platelet aggregates according to size into small, medium, or large aggregates [14]. Platelet aggregation was induced by 5 and 20 μM ADP. Inhibition of platelet aggregation induced by ADP was expressed as absolute reduction of maximal platelet aggregation (baseline response – post-administration response). Phosphorylation of vasodilator-stimulated phosphoprotein (VASP) was measured according to the manufacturer’s recommendation (PLT VASP/P2Y12; Biocytex Inc., Marseille, France), and quantified by flow cytometry (FACSAria II; BD Biosciences, San Jose, CA) and expressed as the platelet reactivity index (PRI).

To measure active metabolites of thienopyridines, blood was collected in a tube containing EDTA at 0.5 and 3 h after the loading dose, and 0.5 mol/L 3′-methoxyphenacyl bromide was immediately added to stabilize the active metabolite. The concentrations of active metabolites of prasugrel (R-138727) and clopidogrel (R-130964) were measured using liquid chromatography with tandem mass spectrometry at LSI Medience Co., Ltd. (Tokyo, Japan).

### Statistical analysis

A sample size of 17 patients in each arm was required to provide at least 80% power to detect a 20% difference in the VASP-PRI at 3 h after the LD with a standard deviation of 20% at a two-tailed significance level of 0.05. Previous reports that compared the pharmacodynamics of clopidogrel with those of prasugrel reached the conclusion that 17 to 24 patients were required in each arm [15, 16].

Statistical analyses were performed using SPSS version 23.0 software (SPSS Inc., Chicago, IL). Normality was first assessed using the Kolmogorov–Smirnov test. Normally distributed variables were compared by the Student’s *t* test, while non-normally distributed variables were compared by the Mann–Whitney *U* test. Categorical variables are expressed as frequency, and were compared using the chi-square test or Fisher’s exact test. Differences in the time course of platelet reactivity (VASP-PRI, change in maximum light transmission, area under the curve of light intensity) between groups were determined by repeated measures ANOVA. All significance tests were two-tailed and *P* < 0.05 was considered statistically significant.

## Results

### VASP phosphorylation and platelet aggregation

**Fig. 2:**
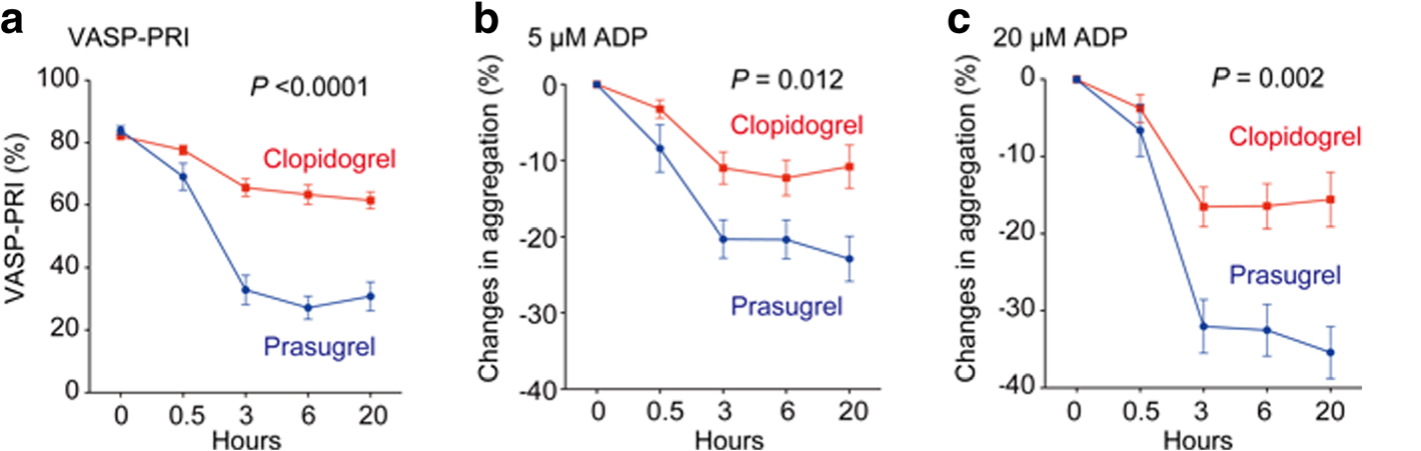
Comparison of platelet function after the loading dose (LD) between clopidogrel and prasugrel. **a** The vasodilator-stimulated phosphoprotein-platelet reactivity index (VASP-PRI) was measured at the indicated time points after the LD. **b**, **c** Platelets in platelet-rich plasma obtained at the indicated time points were stimulated with 5 μM adenosine diphosphate (ADP) (**b**) or 20 μM ADP (**c**). The absolute change in maximal platelet aggregation as assessed by light transmission (baseline – an indicated point) was calculated. Blue lines and red lines represent the prasugrel LD (20 mg) and clopidogrel LD (300 mg), respectively. Values are means ± SEM. Differences in the time course between groups were determined by repeated measures ANOVA

Forty patients were randomly assigned to either prasugrel (*n* = 20) or clopidogrel (*n* = 20) (Fig. 1). Baseline characteristics are shown in Table 1. Fig. 2 shows the results of platelet P2Y_12_ inhibition as assessed by the VASP-PRI and platelet aggregation, which was measured by light transmission after the LD. Baseline VASP-PRI and maximal platelet aggregation induced by ADP were similar in the prasugrel and clopidogrel groups (VASP-PRI: 84.0 ± 6.4 vs. 82.1 ± 4.4, *P* = 0.24; % of light transmission [ADP 20 μM]: 65.0 ± 11.2 vs. 67.5 ± 9.4, *P* = 0.58). Patients who were treated with prasugrel showed significant inhibition of platelet aggregation and the VASP-PRI at 3 h after administration (Fig. 2). The difference between the two drugs was consistent thereafter.

**Table 1:** Baseline Characteristics of the Study Population

	Prasugrel	Clopidogrel	
Variable	(*n* = 20)	(*n* = 20)	*p*-Value
Age, yrs	61.1 ± 11.0	66.1 ± 11.2	0.16
Male, n (%)	17 (85)	13 (65)	0.14
Body mass index, kg/m^2^	27.4 ± 3.6	26.3 ± 4.3	0.37
Current Smoker, n (%)	7 (35)	4 (20)	0.24
Hypertension, n (%)	13 (65)	17 (85)	0.14
Dyslipidemia, n (%)	15 (75)	11 (55)	0.19
Diabetes, n (%)	8 (40)	7 (35)	0.74
SDB (ODI 3%), n (%)	6 (30)	6 (30)	1.00
Previous MI, n (%)	0 (0)	0 (0)	1.0
Previous AP, n (%)	2 (10)	1 (5)	0.55
Previous PCI, n (%)	2 (10)	1 (5)	0.55
Previous CABG, n (%)	0 (0)	0 (0)	1.0
Previous stroke, n (%)	1 (5)	1 (5)	1.0
PAD, n (%)	0 (0)	0 (0)	1.0
Family History of CAD, n (%)	8 (40)	6 (30)	0.51
LVEF, %	69.9 ± 6.3	69.4 ± 6.2	0.74
eGFR, ml/min/1.73m^2^	65.8 ± 18.5	68.2 ± 17.2	0.68
Platelet counts, ×10^3^/μL	21.0 ± 5.9	20.0 ± 4.2	0.54
Medications			
Aspirin, n (%)	20 (100)	20 (100)	1.00
PPI, n (%)	15 (75)	11 (55)	0.19
OAD, n (%)	8 (40)	4 (20)	0.17
Insulin therapy, n (%)	0 (0)	1 (5)	0.31
Statins, n (%)	15 (75)	11 (55)	0.19
Nitrates, n (%)	6 (30)	3 (15)	0.26
CCB, n (%)	9 (45)	10 (50)	0.75
ACEIs/ ARBs, n (%)	11 (55)	11 (55)	1.00
Beta blockers, n (%)	15 (75)	12 (60)	0.31

Values are mean ± SD or n (%)

*ACEIs* Angiotensin converting enzyme inhibitors, *AP* Angina pectoris, *ARBs* Angiotensin receptor blockers, *CABG* Coronary artery bypass surgery, *CAD* Coronary artery disease, *CCB* Calcium channel blockers, *LVEF* Left ventricular ejection fraction, *MI* Myocardial infarction, OAD Oral antidiabetic drug, *PCI* Percutaneous coronary intervention, *PPI* Proton pump inhibitor, *SDB* Sleep-disordered breathing

**Fig. 3:**
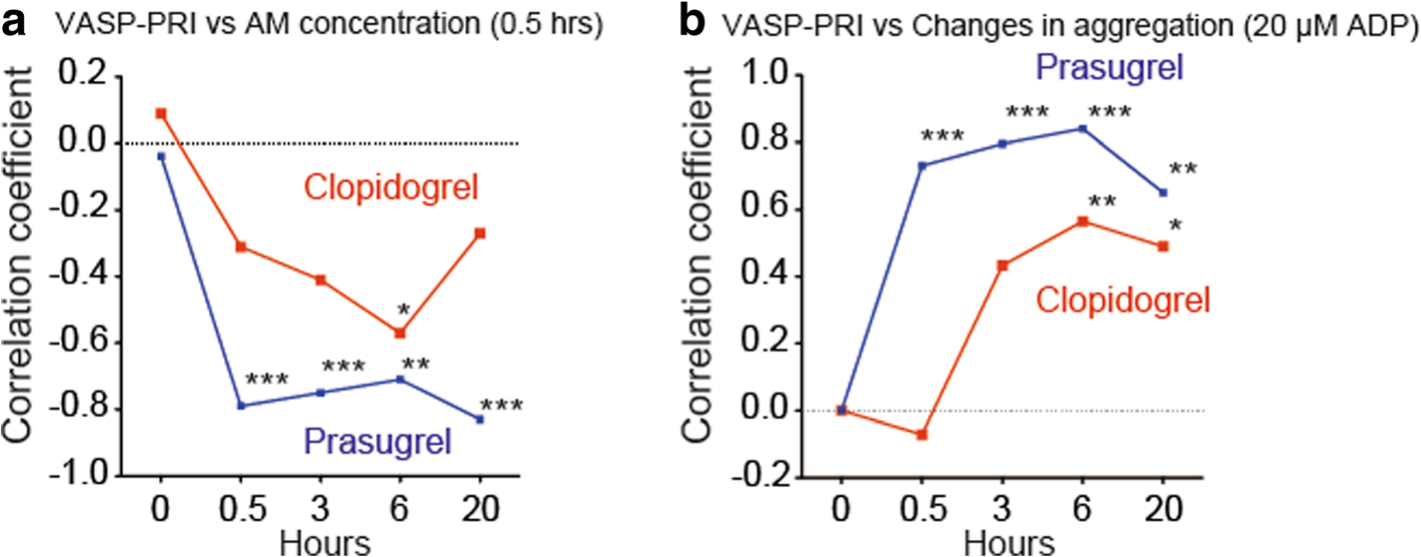
Changes in the correlation coefficient between variables. **a** Changes in Spearman’s correlation coefficient according to the time course between the vasodilator-stimulated phosphoprotein-platelet reactivity index (VASP-PRI) and active metabolite concentrations (AM) measured at 30 min after the LD. **b** Changes in Spearman’s correlation coefficient according to the time course between the VASP-PRI and maximal platelet aggregation induced by 20 μM adenosine diphosphate (ADP). Blue lines and red lines represent the LD of prasugrel (20 mg) and that of clopidogrel (300 mg), respectively. *P < 0.05; **P < 0.01; ***P < 0.001

To examine the timing of P2Y_12_ inhibition by active metabolites, we compared the correlation of the VASP-PRI with active metabolite concentrations. Active metabolite concentrations that were measured at 30 min were highly correlated with the VASP-PRI in prasugrel-treated patients (Fig. 3a). A similar high association was also found between the VASP-PRI and platelet aggregation (Fig. 3b). However, these associations appeared to be weak and delayed in clopidogrel-treated patients (Fig. 3).

**Fig. 4:**
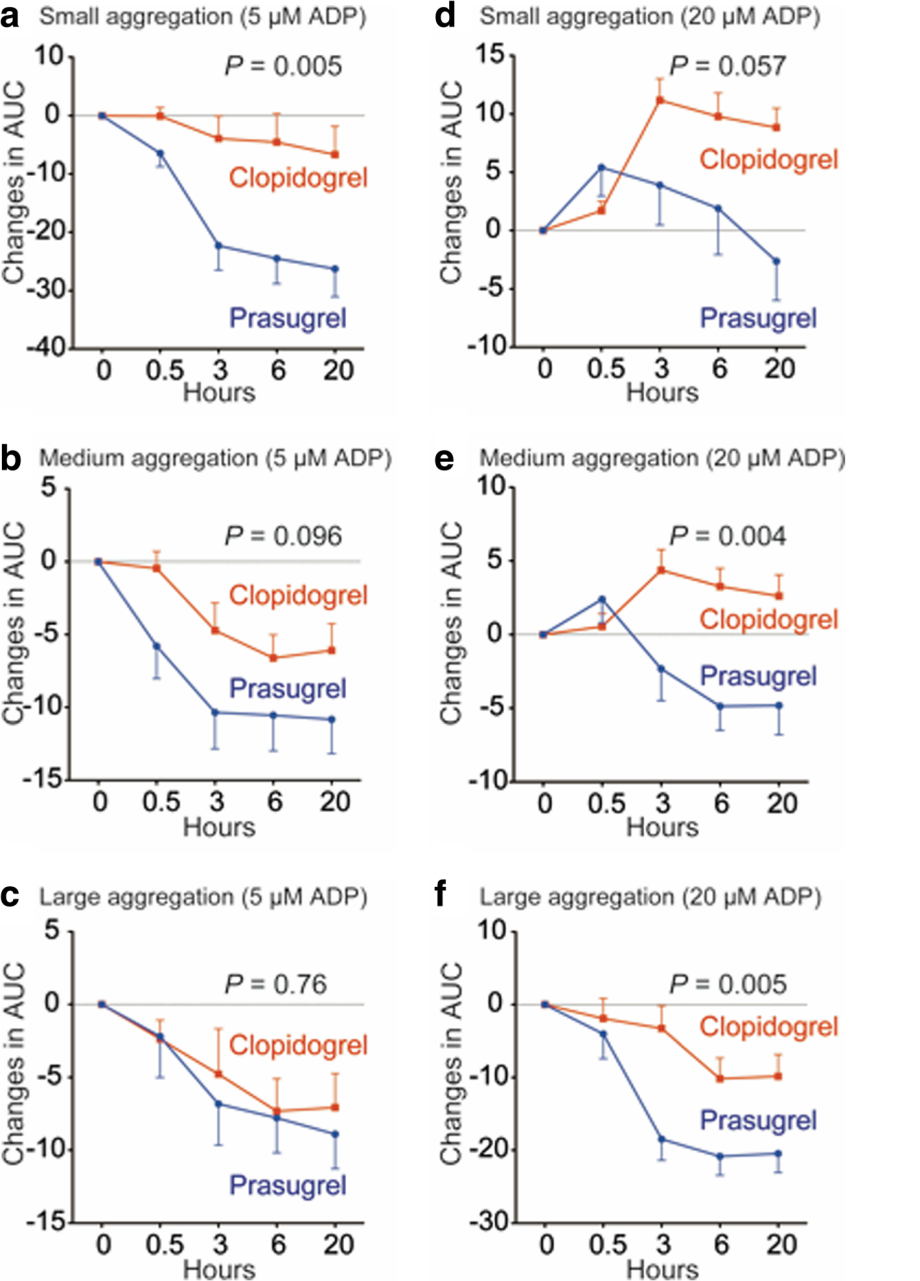
Platelet aggregation as assessed by the laser light scattering method after the loading dose (LD). Platelets in platelet-rich plasma obtained at the indicated time points after the LD were stimulated with 5 μM adenosine diphosphate (ADP) (**a**–**c**) or 20 μM ADP (**d**–**f**). Small (**a**, **d**), medium (**b**, **e**), and large (**c**, **f**) platelet aggregate formation was measured by the laser light scattering method. This formation is expressed as changes in the area under curve (AUC) of each platelet aggregate number during 5 min ( x 10^6^ V/min). Blue lines and red lines represent the LD of prasugrel (20 mg) and that of clopidogrel (300 mg), respectively. Values are means ± SEM. Differences in the time course between groups were determined by repeated measures ANOVA

We next compared the profiles of platelet aggregates by the laser light scattering method. When platelets were stimulated with a low concentration of ADP (5 μM), formation of large aggregates was similarly inhibited in prasugrel and clopidogrel treatment (Fig. 4c). Formation of small aggregates was abolished only by prasugrel treatment (Fig. 4a). Inhibition of P2Y_12_ signalling by clopidogrel appeared to be incomplete because clopidogrel treatment failed to inhibit platelet aggregate formation that was induced by higher concentrations of ADP (20 μM; Fig. 4d–f). Clopidogrel treatment increased formation of medium and small platelet aggregates after platelet stimulation (Fig. 4d, e).

### Differences in determinants of drug efficacy after the LD

To determine the factors that are associated with the inter-individual variability of drug efficacy in both drugs, we analysed the correlation of the VASP-PRI with patients’ characteristics and laboratory data after the LD. The presence of diabetes mellitus and active metabolite concentrations were consistently correlated with the VASP-PRI in prasugrel treatment (Table 2). Diabetes was only independent variable to associate with VASP-PRI in the multiple regression analysis adjusted for age, gender, body mass index, dyslipidemia, hypertension, sleep disordered breathing, platelet counts and PPI use (VASP-PRI 3 h; *R*^2^ = 0.93, *P* < 0.001, 6 h; *R*^2^ = 0.92, *P* = 0.001, 20 h; *R*^2^ = 0.86, *P* = 0.006). Although diabetes did not affect VASP-PRI in clopidogrel-treated patients, sleep-disordered breathing assessed by the oxygen desaturation index showed a positive correlation. A positive association between the VASP-PRI and active metabolite concentrations was observed only at 6 h after administration in the case of clopidogrel (Table 2).

**Table 2:** Correlation of various factors with VASP-PRI

	Prasugrel					Clopidogrel				
	*R*									
Variable	pre	0.5 h	3 h	6 h	20 h	pre	0.5 h	3 h	6 h	20 h
Age, yrs	−0.28	−0.16	−0.33	−0.31	−0.27	0.14	−0.32	−0.18	−0.30	−0.13
Male	−0.29	−0.24	−0.13	−0.04	0.03	0.12	0.28	0.16	0.40	0.28
Body weight, kg	0.01	0.22	0.23	0.29	0.41	0.13	0.27	0.22	0.32	0.16
Body mass index, kg/m^2^	−0.02	0.23	0.25	0.27	0.38	0.26	0.19	0.13	0.26	0.08
Body surface area, m^2^	−0.06	0.16	0.21	0.28	0.39	0.14	0.40	0.21	0.37	0.17
Current Smoker	−0.02	0.32	0.14	0.26	0.20	0.13	0.31	0.33	0.41	0.41
Alcohol	0.06	−0.06	−0.14	0.10	0.0	0.02	0.36	0.22	0.38	0.1
Hypertension	0.38	0.20	0.28	0.38	0.30	0.46*	0.30	0.16	0.01	0.09
Dyslipidemia	0.02	0.44	0.22	0.50*	0.37	−0.17	−0.23	−0.12	−0.05	−0.11
Diabetes	0.14	0.62**	0.70***	0.64**	0.68***	−0.20	−0.17	−0.08	−0.12	−0.23
eGFR, ml/min/1.73m^2^	0.14	0.30	0.39	0.57*	0.44	−0.02	0.19	0.34	0.32	0.37
Platelet counts, ×10^3^/μL	0.43	0.24	0.52*	0.53*	0.39	−0.08	−0.16	−0.10	−0.20	−0.29
Proton pump inhibitor	−0.17	0.14	0.07	0.21	0.35	0.06	0.25	0.17	0.27	0.24
Concentration (0.5 h)	−0.04	−0.79***	−0.75***	−0.71**	−0.83***	0.09	−0.31	−0.41	−0.57*	−0.27
ODI3%	0.15	−0.16	0.18	0.05	0.03	0.12	0.50*	0.48*	0.49*	0.44*

Analysis was performed using the spearman’s correlation coefficient. *P < 0.05; **P < 0.01; ***P < 0.001. ODI = oxygen desaturation index

**Fig. 5:**
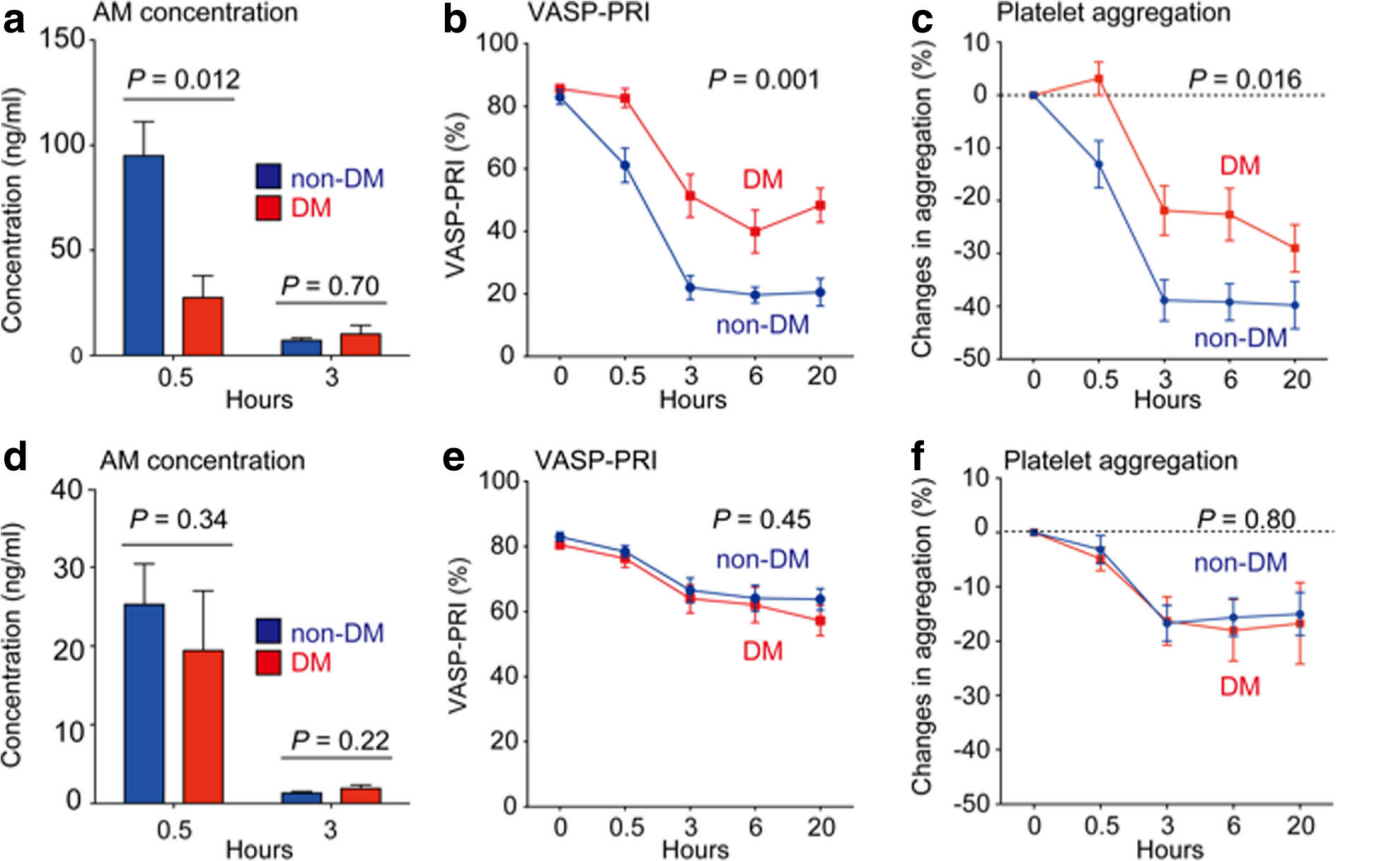
Differential effect of diabetes on active metabolite formation and platelet function between prasugrel and clopidogrel. **a**, **d** Plasma active metabolite (AM) levels after the loading dose (LD) of prasugrel (**a**) or clopidogrel (**d**) were measured at 0.5 and 3 h after treatment in patients without diabetes (blue bar) and those with diabetes (red bar). Values are means ± SEM. Statistical significance was determined using the Mann–Whitney *U* test. **b**, **e** The vasodilator-stimulated phosphoprotein-platelet reactivity index (VASP-PRI) was measured at the indicated time points after the LD of prasugrel (**b**) or clopidogrel (**e**). Blue lines and red lines represent patients without diabetes and those with diabetes, respectively. Values are means ± SEM. Differences in the time course between groups were determined by repeated measures ANOVA. **c**, **f** Platelets in platelet-rich plasma obtained at the indicated time points were stimulated with 20 μM adenosine diphosphate (ADP). The absolute change in maximal platelet aggregation after the LD of prasugrel (**c**) or clopidogrel (**f**) as assessed by light transmission (base line – an indicated point) was calculated. Blue lines and red lines represent patients without diabetes and those with diabetes, respectively. Values are means ± SEM. Differences in the time course between groups were determined by repeated measures ANOVA

To determine the importance of diabetes on drug metabolism and inhibition of platelet aggregation, we compared active metabolite concentrations, the VASP-PRI, and inhibition of platelet aggregation induced by 20 μM ADP in patients with diabetes and those without diabetes. Eight patients in the prasugrel treatment group and seven in the clopidogrel treatment group were previously diagnosed with diabetes. Patients with diabetes had significantly lower levels of active metabolites with prasugrel treatment but not with clopidogrel treatment (Fig. 5a). Reflecting the lower levels of active metabolites, inhibition of the VASP-PRI and platelet aggregation in diabetes was attenuated in prasugrel treatment (Fig. 5b, c). In patients without diabetes, the VASP-PRI was already reduced 30 min after the prasugrel LD (non-diabetes vs. diabetes: 61.1% ± 5.5% vs. 82.7% ± 3.0%, *P* = 0.009). These differences were not observed in clopidogrel treatment. This finding suggests that diabetes has the potential to specifically interfere with pharmacokinetics of prasugrel after a LD.

## Discussion

Application of the new generation of P2Y_12_ inhibitor has improved clinical outcomes after PCI in several randomized, clinical trials [7, 17, 18] . The drug efficacy of thienopyridines affects not only the prognosis but also bleeding complications. Therefore, factors that affect the efficacy of thienopyridine to adequately inhibit P2Y_12_ signalling should be identified in each individual. In the current study, we found reduced levels of active metabolites in diabetes after a LD of prasugrel but not clopidogrel. Further, incomplete inhibition of P2Y_12_ by clopidogrel resulted in an increase in medium and small platelet aggregate formation, which could be overcome by prasugrel.

The most important finding in our study is the differential effect of diabetes on the efficacy between prasugrel and clopidogrel. Increase in active metabolites of prasugrel might be inhibited by the presence of diabetes, resulting in high on-treatment platelet reactivity. Numerous studies have suggested that patients with diabetes have increased platelet reactivity and a reduced platelet response to clopidogrel [19, 20]. However, our study suggests a differential effect of diabetes on antiplatelet effects of thienopyridines. This phenomenon is probably due to variance in determining active metabolite concertation. Most absorbed clopidogrel is hydrolysed by esterase, and only 15% is transformed into the active metabolite by two CYP oxidation steps [5]. Therefore, the *CYP2C19* loss of function polymorphism has a strong effect to determine active metabolite concentration of clopidogrel. On the other hand, absorbed prasugrel is completely converted by esterase to an intermediate metabolite and then is easily oxidized independently of the *CYP2C19* polymorphism. This suggests that the absorbent process in the intestine may predominantly affect active metabolite concentration. Gastroenteropathy manifesting in delayed gastric emptying and constipation causes significant morbidity in patients with diabetes [21]. Gastrointestinal neuromuscular dysfunction in diabetes may reduce absorption of prasugrel after ingestion. Recently, a study reported that crushed prasugrel administration resulted in faster drug absorption and more prompt and potent antiplatelet effects [22]. Such a strategy may overcome the delayed absorption of prasugrel in diabetes.

We also found differences in the platelet aggregation process under treatment with thienopyridines ex vivo. Treatment with clopidogrel inhibited large platelet aggregation but increased small and medium platelet aggregation by stimulation with high ADP concentrations. Platelet aggregation consists of two phases as follows. Formation of small-sized aggregates is followed by the phase of large aggregate formation with a concomitant decrease in the number of small aggregates [23]. P2Y_12_ signalling is important for maintenance of GPIIb/IIIa (integrin αIIbβ3) activation, which causes stable platelet aggregation to interact with fibrinogen [24]. This suggests that incomplete inhibition of P2Y_12_ results in an increase in small and medium aggregates, despite inhibition of large aggregates. One reason why prasugrel reduces coronary events, including stent thrombosis, compared with clopidogrel, may be that this drug effectively abolishes formation of small aggregates. However, the increase in bleeding complications by strong inhibition of P2Y_12_ should be considered. Because patients with P2Y_12_ mutations demonstrate severe bleeding diathesis [25, 26], complete inhibition of P2Y_12_ signalling by thienopyridines causes a tendency for severe bleeding. To identify the optimal range of P2Y_12_ inhibition, evaluation of the ratio among small, medium, and large platelet aggregate formation after ADP stimulation might become an index of adequate P2Y_12_ inhibition in thienopyridine-treated patients.

We demonstrated the effect of diabetes on prasugrel metabolism, while previous reports showed inconsistent results for the role of diabetes [9, 27]. One possible explanation of this discrepancy between studies is the introduction of a lower LD in Japanese patients. This relative reduction in dose may lead to malabsorption of drugs in diabetes. There are several reasons to administrate low-dose prasugrel in Japan as follows: 1) bleeding complications, including cerebral haemorrhage, are more frequent in Japanese [28]; 2) the inhibitory effect on platelet aggregation is more potent [29]; and 3) a previous study was conducted at a lower dose and effectively inhibited platelet aggregation [16]. Further, we could not find any association of the efficacy of clopidogrel with diabetes, although a number of reports have described its relationship [20, 30]. The high ratio of poor and intermediate metabolizers of clopidogrel in Japanese might have reduced the importance of diabetes in our study. We found that the presence of sleep-disordered breathing was significantly correlated with the VASP-PRI in patients who had clopidogrel treatment. Because of the poor correlation among drug concentrations, the VASP-PRI, and platelet aggregation in patients who have clopidogrel, the cAMP signalling pathway, other than P2Y_12_, may be modified by the presence of sleep-disordered breathing.

### Study limitations

This study has some limitations. First, we applied a low LD of prasugrel (20 mg) compared with that in Western countries (60 mg). Our study included a relatively small number of patients, and did not include patients with acute coronary syndrome. Therefore, our findings may not be generalizable to the condition where prasugrel is indicated in Western countries. Additionally, we could not conclude that a higher LD could overcome defects in drug metabolism in diabetes. Finally, we could not assess bleeding and thrombotic events associated with antiplatelet therapy.

## Conclusions

This study shows the differential effect of diabetes on antiplatelet activity between clopidogrel and prasugrel. We speculated that absorbed process of prasugrel, but not CYP metabolism, affected active metabolite concentration of prasugrel in diabetes. Further, prasugrel efficiently abolishes residual P2Y_12_ signalling that leads to small platelet aggregation, but these small aggregates are not inhibited by clopidogrel. To efficiently apply dual antiplatelet therapy with potent P2Y_12_ blockers, including prasugrel and ticagrelor, further studies are required to understand their optical range for inhibiting P2Y_12_ signalling.

## Abbreviations

ADP: Adenosine diphosphate
AM: Active metabolites
CT: Computed tomography
CYP: Cytochrome P450
LD: Loading dose
PCI: Percutaneous coronary intervention
PRI: Platelet reactivity index
VASP: Vasodilator stimulated phosphoprotein
